# A “growth mindset of mental health” intervention increases the likelihood of initiating self-help on a mental health app: a randomized controlled trial

**DOI:** 10.1038/s41746-026-03322-w

**Published:** 2026-10-13

**Authors:** Sean H. Y. Toh, E. J. Horberg, Feodora R. Kosasih, Oliver Sündermann, Paul A. O’Keefe

**Affiliations:** 1 Department of Psychology, Harvard University, Cambridge, MA USA; 2 Department of Management, University of Exeter Business School, Exeter, UK; 3 Intellect Pte Ltd, Singapore, Singapore

**Keywords:** Diseases, Health care, Psychology, Psychology

## Abstract

Mental health apps hold potential to improve access to treatment, but engagement remains low. One barrier may be the belief that mental health cannot be meaningfully improved. In a randomized controlled trial (*N* = 252), we examined whether a brief *growth-mindset-of-mental-health* intervention—portraying mental health as improvable, not fixed—can initiate mental health app engagement among people with mental health needs. Participants completed baseline well-being measures (stress, anxiety and depression, and positive mental well-being) and were randomly assigned to the growth-mindset-of-mental-health or active control condition. Over the subsequent three months, we tracked whether they re-engaged with the app overall (i.e., completed at least a second self-help feature), and with features categorized as low-, moderate-, and high-intensity. Analyses revealed that participants in the growth-mindset condition (vs. control) with poorer baseline mental well-being (i.e., higher stress, anxiety and depressive symptoms, and lower positive mental well-being) were significantly more likely to show repeat app engagement. Moreover, those with higher anxiety and depressive symptoms were more likely to show repeat engagement with moderate-intensity features, while those experiencing higher stress and lower positive mental well-being were more likely to do so with both low- and moderate-intensity features. Together, these findings demonstrate the benefits of the growth-mindset intervention for successful initiation of self-help on a mental health app. Trial Registration: The trial was registered on ClinicalTrials.gov (Identifier: NCT07071311) on July 25, 2025.

## Introduction

Many people with mental health problems do not seek, access, or sustain professional treatment^1^. The treatment gap, defined as the discrepancy between individuals in need of care and those who receive treatment, remains alarmingly wide, with estimates ranging from 40% in high-income countries to as high as 85% in low- and middle-income countries^2,3^. Multiple factors contribute to this treatment gap, including stigma, financial constraints, and poor mental health literacy in high-income countries^4^, with low- and middle-income countries also facing shortages of mental health professionals and treatment facilities^5^.

In recent years, mental health applications (MHAs) have emerged as promising and scalable tools to bridge this gap^6–8^. Recent systematic reviews indicate a growing number of MHAs incorporating evidence-based strategies, such as cognitive behavioral therapy (CBT) and mindfulness, which have been shown to effectively reduce the severity of common mental health problems (e.g., stress, anxiety, and depression)^9,10^. These apps have demonstrated efficacy in randomized controlled trials (RCTs) and across various age groups, showing that they can equip individuals struggling with poor mental well-being with skills to manage their mental health effectively. Many of these MHAs are also either free or low-cost, widely available to anyone with a smartphone, and can be used privately and on-the-go^11^. Taken together, these evidence-based MHAs represent a scalable avenue with the potential to deliver reliable mental health support beyond traditional clinical settings.

Despite their potential, continuous engagement with MHA features remains a longstanding challenge^12,13^. Most users discontinue their use after minimal interaction^14–18^, which substantially limits the potential benefits of these features. This pattern is evident in a systematic review by Fleming et al. (2018) examining real-world uptake of digital mental health treatments, who found that up to 80% of users logged in only once after downloading the mobile app and did not return to complete another activity^19^. These findings were supported by an earlier review assessing the engagement rates of 12 publicly available MHAs, where the most common pattern of use was launching the app only once^20^.

These trends are evident even among widely used and empirically supported apps. For instance, in Australia’s most widely adopted CBT-based mental health app (MoodGYM), which has demonstrated efficacy across RCTs in reducing mental health symptoms, only 7.0% to 16.0% of users returned for further engagement^21–25^. Similarly, in apps that have promoted positive well-being, such as Happify Health, only 20.6% of users voluntarily returned to complete a second activity after their initial interaction^26^.

Efforts to improve engagement have often focused on technical features, such as enhancing user-friendliness, personalizing feedback, customizing self-help activities, and implementing features that boost social connection^27,28^. However, MHAs that have incorporated such features have not consistently demonstrated higher user engagement^13,29^. In addition to technical enhancements, participant characteristics such as demographic factors, baseline symptom severity, and familiarity with technology use have also been examined, but no single predictor has reliably predicted higher app engagement metrics across studies, and most proposed predictors show only small effects when examined in isolation^15^. In a comprehensive review of predictors of engagement with digital interventions, Linardon and Fuller-Tyszkiewicz (2024) found that engagement was best predicted by the aggregation of many small effects across multiple predictors. Collectively, these findings highlight that strategies centered on enhancing app features or relying on unmodifiable participant characteristics have had limited success in sustaining engagement with MHAs^15,30^. This is particularly concerning for people struggling with their mental health, as repeated engagement with MHA features has often been associated with greater mental health benefits (i.e., stronger dose–response relationships^31–33^), particularly among individuals with higher baseline symptom severity^34^.

Beyond considerations of app design and participant characteristics, psychological or attitudinal factors have been shown to significantly influence whether individuals pursue mental health support for themselves, yet these factors remain largely understudied in the context of MHA engagement^35^. One such factor is a person’s mindset, which can shape behavior in meaningful ways. Decades of research on fixed and growth mindsets demonstrates that one’s beliefs about the malleability of personal attributes can profoundly influence a wide array of behaviors^36–39^. A *fixed mindset* is the belief that a particular attribute (e.g., intelligence, personality, willpower, interest, opportunities) is relatively unchangeable. In contrast, a *growth mindset* reflects the belief that an attribute is malleable and, therefore, can be developed and improved^40–42^. For instance, middle school students with a growth mindset of intelligence—the belief that intelligence is malleable—showed significant academic gains over the following two years compared to those with a fixed mindset^41,43,44^. As another example, adults who viewed their shyness as a changeable trait were more likely to actively engage in social interactions, and adopt behaviors that mitigated the negative consequences of their shyness, relative to those who believed their shyness was relatively unchangeable^45^.

Critically, this perspective has been extended to beliefs about the growth versus fixed nature of attributes related to mental health. For example, people who endorsed a growth mindset of emotion—believing that emotions are malleable and can be regulated and improved—were more likely to adopt adaptive coping strategies, such as actively reappraising negative thoughts^46^. These strategies have been linked to positive outcomes, including greater subjective well-being and improved satisfaction in interpersonal relationships^47^. On the other hand, those with a fixed mindset of emotion relied more on avoidance-based strategies, such as suppression, substance use, distraction, or disengagement, all of which have been linked to greater psychological distress^48–50^. Additionally, Schroder et al. (2015) found that undergraduate students with a growth mindset about anxiety preferred seeking psychotherapy as their primary treatment, whereas those with a fixed mindset of anxiety favored medication-based treatments, attributing their mental health difficulties to uncontrollable genetic factors and feelings of helplessness^48^. Participants with higher levels of positive mental well-being also tended to endorse a stronger growth mindset about mental well-being at baseline (e.g., greater happiness, more satisfying interpersonal relationships)^47^, positive affect and overall flourishing^51,52^ were positively correlated with growth mindset of well-being measures (*r* = 0.20 to 0.52). In contrast, those who reported more severe mental health symptoms, including psychological distress^53^, depression and anxiety (*r* = −0.37 to −0.49)^53,54^, were more likely to hold fixed beliefs about mental well-being. That is, a fixed mindset may serve as a psychological barrier to engaging in proactive care.

Research has also shown that a growth mindset of well-being can be induced to produce positive outcomes. Howell et al. (2016) experimentally induced a growth mindset of well-being by presenting university students with information about (a) the effectiveness of well-being interventions, (b) the role of attitudes in supporting overall health, and (c) testimonials from older adults who had improved their health. Following these experimental tasks, students reported greater openness to adopting positive lifestyle changes, such as increasing their exercise levels, participating in recreational activities, and engaging with nature^51^. These findings suggest that interventions aimed at fostering a growth mindset about mental health could help increase individuals’ willingness and propensity to engage with self-help treatments to manage their own mental health.

Indeed, growth-mindset interventions have successfully promoted adaptive behaviors. For example, in the education domain, O’Keefe et al. (2023) developed a growth-mindset intervention that helped first-year undergraduate students understand that new academic interests can be cultivated, and are not fixed^55^. Among arts students who initially lacked an affinity for math and science, this intervention boosted subsequent interest and achievement in their required math and science courses, compared to an active control condition (see other examples, refs. ^39,43,56,57^).

In the present study, our primary goal was to test whether a growth-mindset-of-mental-health intervention (promoting the belief that mental health is improvable, not fixed) could boost the likelihood of repeat engagement with MHAs among employees with mental health needs, as compared to an active control group. To examine this, we conducted a two-armed RCT with working adults who had access to an evidence-based MHA, *Intellect*, through their organizations. Intellect is a digital mental health platform that serves as the official mental healthcare provider for various government agencies, insurance companies, healthcare organizations, hospitals, and corporations across Singapore and the Asia-Pacific region. The platform offers a broad range of self-guided mental health activities rooted in evidence-based practices, such as Cognitive Behavioral Therapy (CBT), Mindfulness, Acceptance and Commitment Therapy (ACT), and Positive Psychology, and some of these features have demonstrated efficacy in previous RCTs^58–64^.

After completing baseline measures of well-being, participants were randomly assigned to complete either the growth-mindset-of-mental-health intervention or the active control materials. We then assessed whether they showed *repeat engagement* (i.e., returning to the app to complete at least one additional self-help feature) over the subsequent 3-month period. This outcome was designed to assess successful initiation of engagement, rather than casual, exploratory use^65^. This outcome captures the likelihood of engagement beyond the initial use, and does not assess the frequency, duration, or depth of engagement with the app’s features. If the growth-mindset-of-mental-health intervention boosts the likelihood of repeat MHA engagement, it may suggest that viewing mental well-being as improvable could motivate people to take the important step of initiating engagement with mental health resources.

Our central hypothesis is that, among people with poorer mental well-being at baseline, the growth-mindset-of-mental-health intervention (vs. control) will increase the likelihood of repeat engagement with the app’s self-help features following the intervention. Employees with poorer well-being are not only more likely to view engagement with the MHA as personally relevant, but they are more likely to hold fixed beliefs about mental well-being^66^. As such, they may stand to benefit more from an intervention that instills a growth mindset of mental health, and consequently take more proactive steps to support their mental well-being.

Additionally, in secondary analyses we examined whether, among individuals with lower (but not higher) baseline mental well-being, the growth-mindset-of-mental-health intervention (vs. control) would increase the likelihood of repeat engagement with app features of varying intensities—low, moderate, and high—categorized by the increasing effort and time required to complete them. Low-intensity features included mood or stress tracking; moderate-intensity features included short exercises such as breathing techniques or thought-challenging activities; and high-intensity features included more effortful activities such as guided journaling or structured, psychoeducation programs.

## Results

### Analyses of excluded participants

Out of the 272 participants who enrolled in the study, 136 were randomly assigned to the growth-mindset condition, while 136 were allocated to the active control condition. Among the 136 participants in the intervention group, 11 were excluded due to (a) bot-detected fraudulent responses (3/136, 2.21%), (b) duplicate responses (7/136, 5.15%), as well as (c) hasty responses (< 4 min; 1/136, 0.74%). As for the 136 control group participants, 9 were excluded for similar reasons: (a) bot-detected fraudulent responses (3/136, 2.21%), (b) duplicate responses (1/136, 0.74%), (c) blanks, nonsensical or non-response answers (e.g., “NA,” “Yes,” “Don’t know”) for all writing activities (3/136, 2.21%), and (d) hasty responses (2/136, 1.47%). This exclusion rate did not differ between conditions (*p*s > 0.05). Moreover, independent sample *t*-tests and χ^2^ tests confirmed that excluded participants were not significantly different from our final sample on any demographic or baseline variables (*p*s > 0.05).

### Descriptive analyses

Table 1 presents the descriptive statistics and intercorrelations of study variables for the full sample. Table 2 presents baseline descriptives by condition. No significant differences were found between conditions on the baseline variables (*p*s > 0.05). Baseline growth mindset of mental health also did not differ significantly between the growth-mindset condition (*M* = 4.31, *SD* = 0.841) and the active control condition (*M* = 4.29, *SD* = 0.831) on a 1–6 point scale, *t*(250) = −0.13, *p* = 0.897.

**Table 1:** Descriptive statistics and intercorrelations among baseline variables

Baseline variables	*M*	*SD*	1	2	3	4	5
1. Age	32.48	7.70					
2. Gender	0.450	0.500	−0.031				
3. Anxiety and Depression	7.96	2.53	−0.220^***^	−0.040			
4. Perceived Stress	11.09	2.80	−0.095	−0.050	0.657^***^		
5. Positive Mental Well-being	23.04	4.80	0.060	0.097	−0.572^***^	−0.695^***^	

^*^p < 0.05; ^**^p < 0.01; ^***^p < 0.001. Gender was coded as Male = 0, Female = 1.

**Table 2:** Descriptive statistics for baseline variables by condition

Variables		Growth-mindset condition (*n* = 125)	Active control condition (*n* = 127)	*p*-value
		*M* (*SD*) */ n* (%)	*M* (*SD*) */ n* (%)	
Age		33.01 (8.13)	31.95 (7.24)	0.277
Gender	Female	49 (39.2%)	63 (49.6%)	0.229
	Male	74 (59.2%)	63 (49.6%)	
	Others	2 (1.6%)	1 (0.8%)	
Anxiety and Depressive Symptoms		7.92 (2.43)	8.00 (2.65)	0.803
Perceived Stress		11.0 (2.71)	11.1 (2.89)	0.825
Positive Mental Well-being		22.9 (4.85)	23.2 (4.75)	0.592

Overall, 33.7% of the full sample showed repeat engagement with self-help features during the 3-month follow-up period (29.1% in the active control condition and 38.4% in the growth-mindset condition).

### Primary analysis: overall repeat engagement of self-help features

Table 3 presents the main effects of condition, baseline well-being variables, and their interaction on repeat engagement with any of Intellect’s self-help features. As shown, no significant effects of condition or baseline well-being were observed when the baseline well-being variable was at its mean level. As hypothesized, however, the growth-mindset condition interacted with each of the three baseline well-being variables to predict a significantly higher likelihood of repeat engagement with the self-help features.

**Table 3:** Interaction of baseline well-being and condition on likelihood of repeat engagement of any self-help features

Variables		Outcome: repeat engagement with self-help features (Yes/No)				
		β	*SE*	*p*-value	LLCI	ULCI
Moderator: Anxiety and Depression						
	Constant	−0.798	0.634	0.208	−2.04	0.445
	Condition	0.407	0.276	0.140	−0.133	0.947
	Anxiety and Depression	−0.062	0.077	0.420	−0.212	0.088
	Condition × Anxiety and Depression	**0.234**	**0.110**	**0.033**	**0.018**	**0.450**
Moderator: Perceived Stress						
	Constant	−0.784	0.623	0.209	−2.01	0.438
	Condition	0.410	0.276	0.137	−0.131	0.951
	Perceived Stress	−0.053	0.069	0.447	−0.189	0.083
	Condition × Perceived Stress	**0.207**	**0.100**	**0.039**	**0.010**	**0.403**
Moderator: Positive Mental Well-being						
	Constant	−0.741	0.628	0.239	−1.97	0.491
	Condition^a^	0.381	0.279	0.173	−0.167	0.928
	Positive Mental Well-being	0.036	0.042	0.390	−0.047	0.119
	Condition × Positive Mental Well-being	**−0.149**	**0.059**	**0.013**	**−0.265**	**−0.032**

*LLCI* lower limit of 95% confidence interval, *ULCI* upper limit of 95% confidence interval.

^a^Reference category: Active Control; Age and dummy-coded gender (reference category: “Not Female”) were controlled for in all models.

Bold values indicate statistically significant effects at *p* < 0.05.

**Fig. 1:**
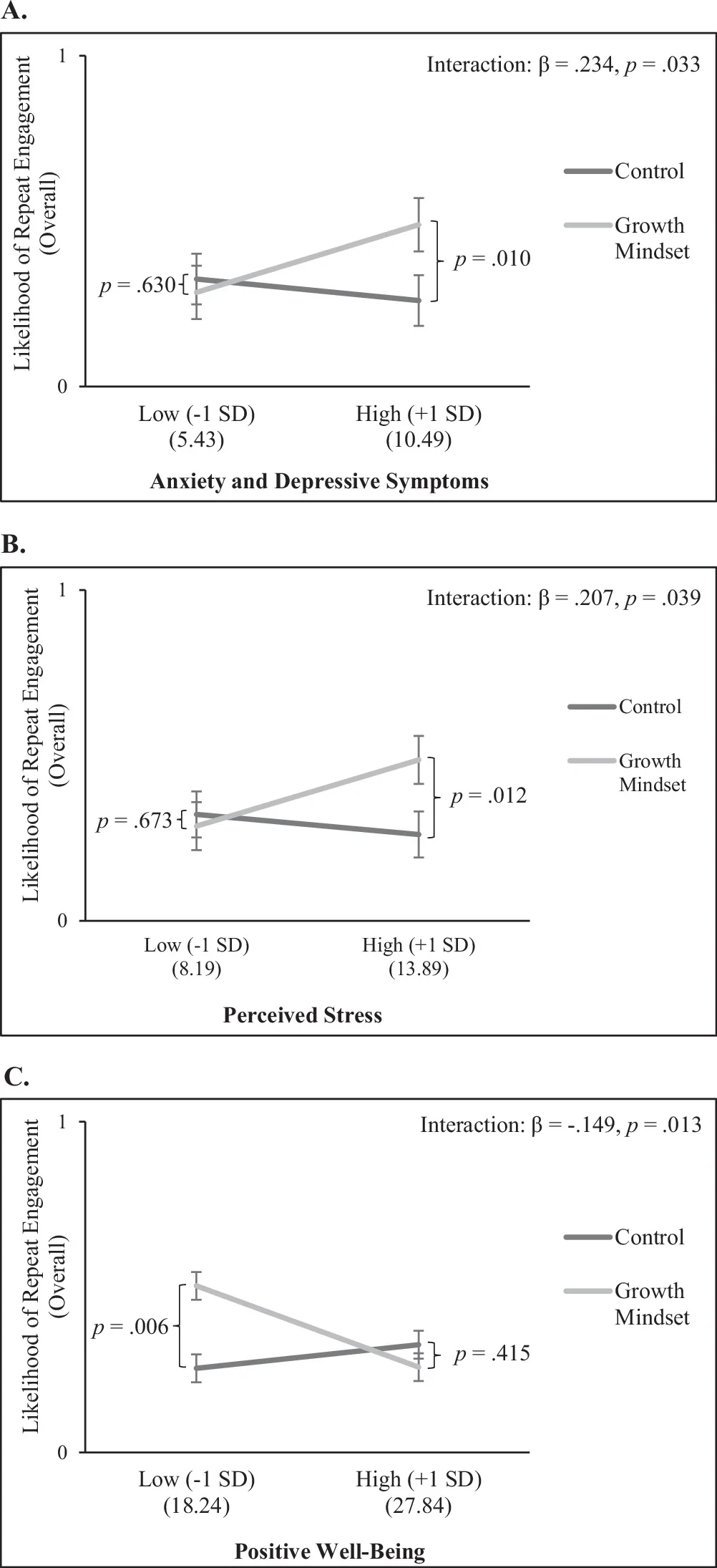
Likelihood of repeat engagement with any self-help features. The panels show the effect as a function of the interaction between condition and **A** baseline anxiety and depressive symptoms, **B** baseline perceived stress, and **C** baseline positive well-being. Raw score values corresponding to −1 *SD* and +1 *SD* for each baseline variable are shown in parentheses.

Figure 1 illustrates the conditional effects of treatment condition on likelihood of repeat engagement at +1 *SD* and −1 *SD* on each baseline well-being measure. These presented a consistent pattern: among participants with lower baseline well-being, those who completed the growth-mindset intervention (vs. control condition) were significantly more likely to return to complete at least another self-help feature. By contrast, there were no differences between conditions among participants with higher baseline well-being. Thus, the intervention was more effective for those experiencing mental health problems.

Specifically, the growth-mindset intervention (vs. active control) significantly increased the likelihood of repeat engagement with self-help features among participants with poorer baseline well-being (i.e., higher anxiety/depression, β = 1.00, *SE* = 0.390, *p* = 0.010, OR = 2.72; higher perceived stress, β = 0.995, *SE* = 0.394, *p* = 0.011, OR = 2.71; lower positive mental well-being, β = 1.10, *SE* = 0.397, *p* = 0.005, OR = 3.01). By contrast, there was no condition difference in the likelihood of repeat engagement among participants who were doing better at baseline (i.e., lower anxiety/depression, β = −0.184, *SE* = 0.393, *p* = 0.640, OR = 0.832; lower perceived stress, β = −0.161, *SE* = 0.393, *p* = 0.682, OR = 0.851; higher positive mental well-being, β = −0.323, *SE* = 0.401, *p* = 0.421, OR = 0.724).

### Secondary analyses: repeat engagement with different types of self-help features

To determine the nature of participants’ repeat engagement, we tested the interaction of treatment condition and baseline well-being measures separately for low-, moderate-, and high-intensity self-help features.

Table 4 presents the results for the likelihood of repeat engagement with low-intensity self-help features. As shown, no significant effects of treatment condition or baseline well-being emerged at mean levels of the baseline well-being variable. However, treatment condition significantly interacted with two of the three baseline well-being variables: perceived stress and positive mental well-being.

**Table 4:** Interaction of baseline well-being and condition on repeat engagement with low-intensity features

Variables		Outcome: repeat engagement with low-intensity features (Yes/No)				
		β	*SE*	*p*-value	LLCI	ULCI
Moderator: Anxiety and Depression						
	Constant	−1.02	0.657	0.119	−2.31	0.264
	Condition^a^	0.242	0.284	0.394	−0.314	0.799
	Anxiety and Depression	−0.057	0.079	0.471	−0.211	0.097
	Condition × Anxiety and Depression	0.180	0.112	0.108	−0.039	0.399
Moderator: Perceived Stress						
	Constant	−1.06	0.648	0.103	−2.33	0.214
	Condition^a^	0.240	0.286	0.402	−0.320	0.800
	Perceived Stress	−0.069	0.072	0.338	−0.210	0.072
	Condition × Perceived Stress	**0.211**	**0.103**	**0.040**	**0.009**	**0.414**
Moderator: Positive Mental Well-being						
	Constant	−1.01	0.651	0.120	−2.29	0.263
	Condition^a^	0.213	0.289	0.461	−0.353	0.779
	Positive Mental Well-being	0.037	0.043	0.398	−0.048	0.122
	Condition × Positive Mental Well-being	**−0.131**	**0.060**	**0.030**	**−0.249**	**−0.013**

*LLCI* lower limit of 95% confidence interval, *ULCI* upper limit of 95% confidence interval.

^a^Reference category: Active Control; Age and dummy-coded gender (reference category: “Not Female”) were controlled for in all models.

Bold values indicate statistically significant effects at *p* < 0.05.

**Fig. 2:**
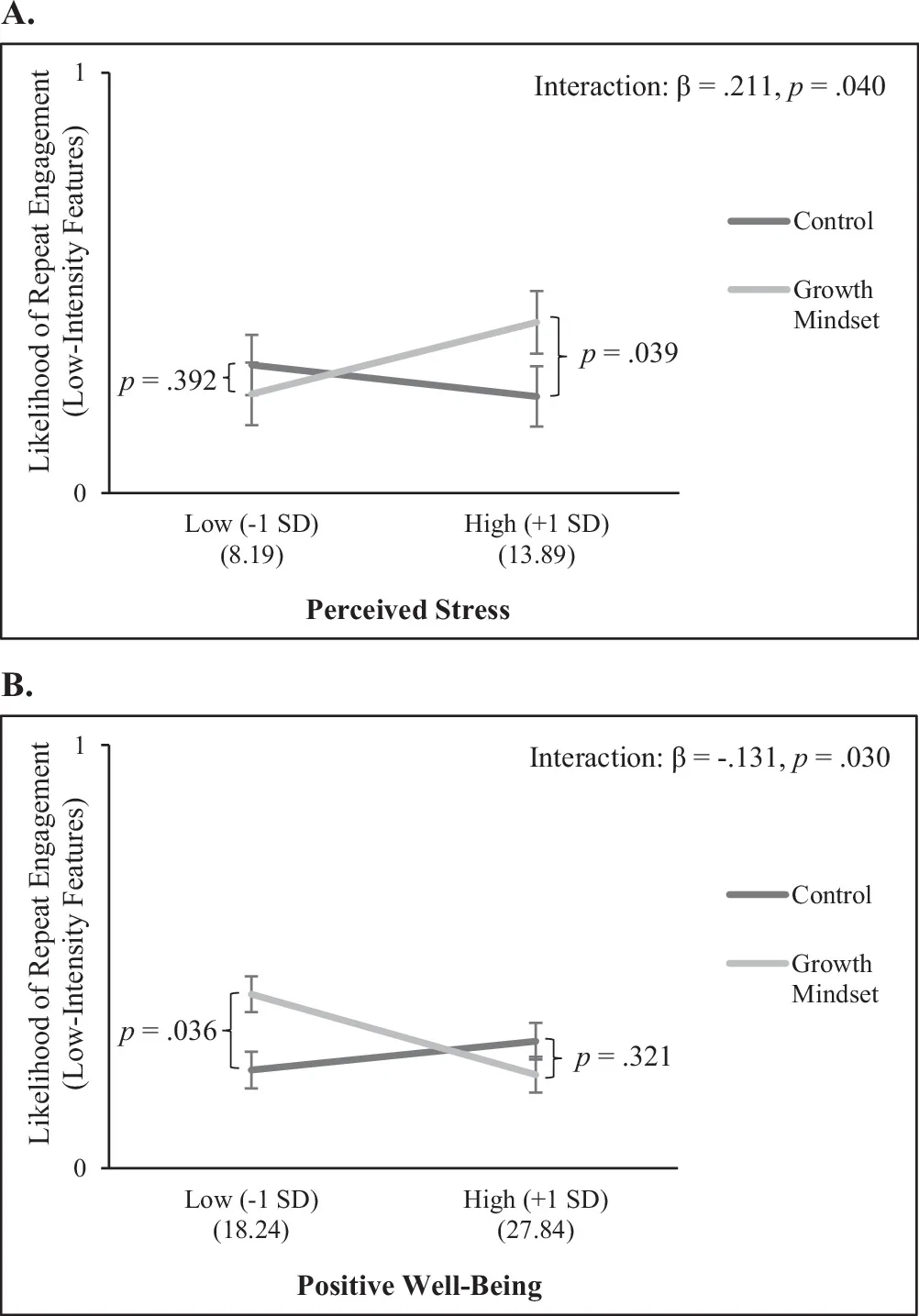
Likelihood of repeat engagement with low-intensity features. The panels show the effect as a function of the interaction between condition and **A** baseline perceived stress, and **B** baseline positive well-being. Raw score values corresponding to −1 *SD* and +1 *SD* for each baseline variable are shown in parentheses.

As shown in Fig. 2, the growth-mindset intervention (vs. control) led to an increased likelihood of repeat engagement with low-intensity features among participants with higher (+ 1 *SD*) baseline perceived stress (β = 0.839, *SE* = 0.405, *p* = 0.038, OR = 2.31) and among those with lower (−1 *SD*) baseline positive mental well-being (β = 0.850, *SE* = 0.404, *p* = 0.035, OR = 2.34). In contrast, there were no condition differences among participants with lower (−1 *SD*) baseline perceived stress (β = −0.345, *SE* = 0.408, *p* = 0.397, OR = 0.708) or higher (+ 1 *SD*) positive mental well-being (β = −0.407, *SE* = 0.414, *p* = 0.325, OR = 0.665).

Table 5 presents the results for moderate intensity self-help features. As shown, treatment condition interacted with all three baseline well-being measures to predict likelihood of repeat engagement with moderate-intensity self-help features.

**Table 5:** Interaction of baseline well-being and condition on repeat engagement with moderate-intensity features

Variables		Outcome: repeat engagement with moderate-intensity features (Yes/No)				
		β	*SE*	*p*-value	LLCI	ULCI
Moderator: Anxiety and Depression						
	Constant	**−1.47**	**0.724**	**0.043**	**−2.89**	**−0.047**
	Condition^a^	0.198	0.312	0.526	−0.412	0.810
	Anxiety and Depression	−0.049	0.085	0.565	−0.216	0.118
	Condition × Anxiety and Depression	**0.274**	**0.122**	**0.025**	**0.035**	**0.514**
Moderator: Perceived Stress						
	Constant	**−1.44**	**0.713**	**0.043**	**−2.84**	**−0.047**
	Condition^a^	0.204	0.312	0.513	−0.408	0.817
	Perceived Stress	−0.029	0.077	0.708	−0.179	0.121
	Condition × Perceived Stress	**0.228**	**0.112**	**0.042**	**0.009**	**0.446**
Moderator: Positive Mental Well-being						
	Constant	−1.32	0.710	0.062	−2.72	0.068
	Condition^a^	0.217	0.313	0.489	−0.397	0.830
	Positive Well-being	0.038	0.047	0.417	−0.054	0.130
	Condition × Positive Well-being	**−0.128**	**0.064**	**0.048**	**−0.254**	**−0.002**

*LLCI* lower limit of 95% confidence interval, *ULCI* upper limit of 95% confidence interval.

^a^Reference category: Active Control; Age and dummy-coded gender (reference category: “Not Female”) were controlled for in all models.

Bold values indicate statistically significant effects at *p* < 0.05.

**Fig. 3:**
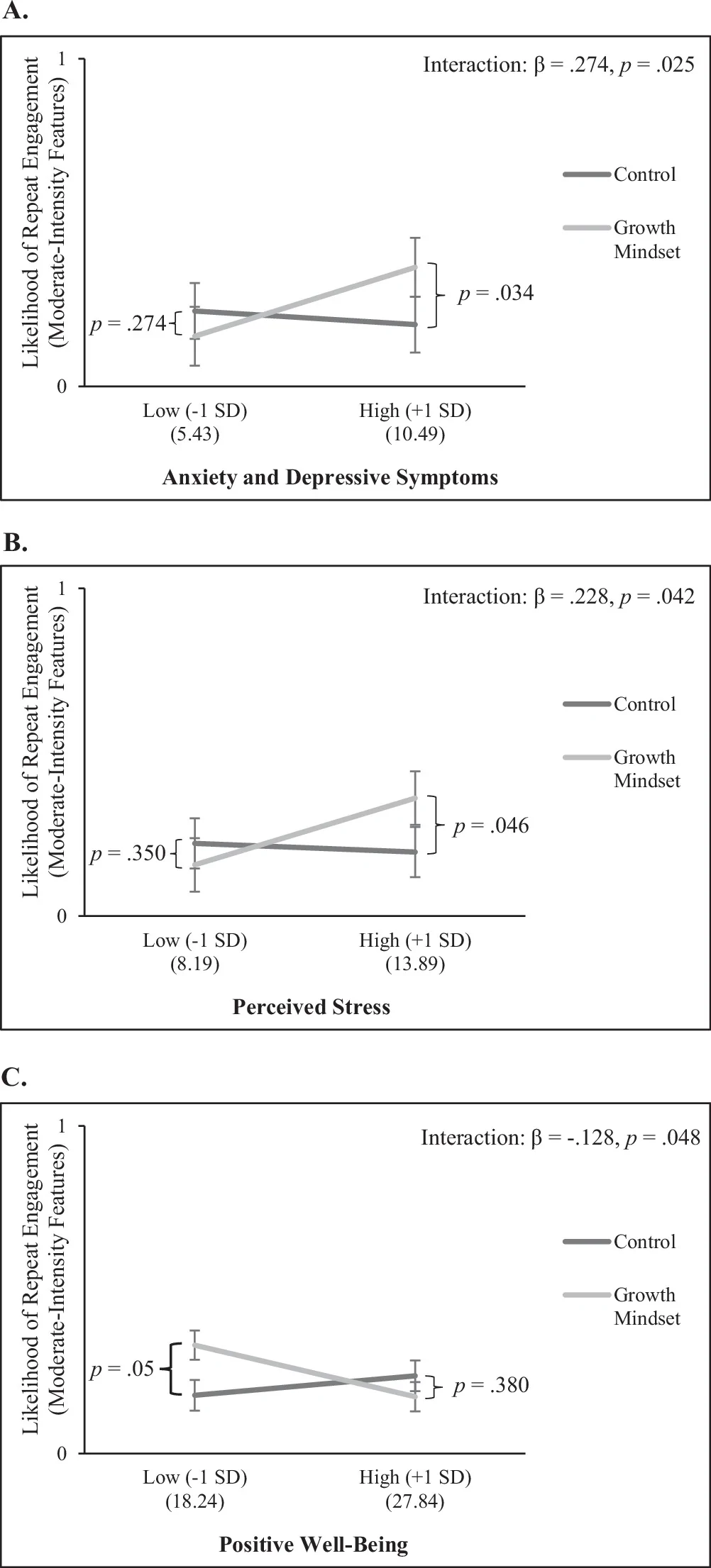
Likelihood of repeat engagement with moderate-intensity features. The panels show the effect as a function of the interaction between condition and **A** baseline anxiety and depressive symptoms, **B** baseline perceived stress, and **C** baseline positive well-being. Raw score values corresponding to −1 *SD* and +1 *SD* for each baseline variable are shown in parentheses.

As illustrated in Fig. 3, the growth-mindset intervention (vs. control) increased the likelihood of repeat engagement with moderate-intensity features for participants with higher (+ 1 *SD*) baseline levels of anxiety and depression (β = 0.895, *SE* = 0.423, *p* = 0.034, OR = 2.45), higher perceived stress (β = 0.849, *SE* = 0.424, *p* = 0.045, OR = 2.34), and lower (−1 *SD*) baseline positive mental well-being (β = 0.837, *SE* = 0.433, *p* = 0.05, OR = 2.31). By contrast, there were no condition differences for participants with lower (−1 *SD*) anxiety and depression (β = −0.494, *SE* = 0.456, *p* = 0.279, OR = 0.610), lower perceived stress (β = −0.425, *SE* = 0.459, *p* = 0.355, OR = 0.654), nor for participants with higher (+ 1 *SD*) positive mental well-being (β = −0.388, *SE* = 0.446, *p* = 0.385, OR = 0.679).

None of the baseline well-being measures interacted with treatment condition to predict the likelihood of repeat engagement with high-intensity self-help features (see Table 6).

**Table 6:** Interaction of condition and baseline well-being on repeat engagement with high-intensity features

Variables		Outcome: repeat engagement with high-intensity features (Yes/No)				
		β	*SE*	*p*-value	LLCI	ULCI
Moderator: Anxiety and Depression						
	Constant	**−1.71**	**0.617**	**0.006**	**−2.91**	**−0.498**
	Condition^a^	0.425	0.263	0.107	−0.091	0.942
	Anxiety and Depression	−0.042	0.072	0.562	−0.183	0.099
	Condition × Anxiety and Depression	0.174	0.105	0.097	−0.031	0.379
Moderator: Perceived Stress						
	Constant	**−1.67**	**0.605**	**0.006**	**−2.85**	**−0.480**
	Condition^a^	0.425	0.263	0.106	−0.090	0.940
	Perceived Stress	−0.016	0.065	0.810	−0.143	0.112
	Condition × Perceived Stress	0.106	0.094	0.262	−0.079	0.291
Moderator: Positive Mental Well-being						
	Constant	**−1.65**	**0.606**	**0.006**	**−2.84**	**−0.463**
	Condition^a^	0.409	0.264	0.121	−0.107	0.926
	Positive Mental Well-being	0.012	0.040	0.763	−0.066	0.090
	Condition × Positive Mental Well-being	−0.080	0.056	0.153	−0.189	0.030

*LLCI* lower limit of 95% confidence interval, *ULCI* upper limit of 95% confidence.

^a^Reference category: Active Control; Age and dummy-coded gender (reference category: “Not Female”) were controlled for in all models.

Bold values indicate statistically significant effects at *p* < 0.05.

## Discussion

The present study investigated whether a brief, single-session growth-mindset-of-mental-health intervention could promote the successful initiation of engagement with self-help features on an evidence-based MHA, among employees with poorer baseline mental well-being. Consistent with our hypotheses, the intervention significantly increased the likelihood of repeat app engagement among participants who began the study with diminished well-being at baseline (i.e., higher levels of anxiety and depression, perceived stress, or lower positive mental well-being), relative to an active control condition. By contrast, and as predicted, the intervention had no significant effect on repeat self-help engagement among participants with relatively high well-being at baseline. Our secondary analyses revealed that these moderation effects were most evident for low- and moderate-intensity app features. For low-intensity features, the intervention condition showed increased repeat engagement among those with higher stress and lower positive well-being at baseline. For moderate-intensity features, the intervention condition showed increased repeat engagement among those with higher anxiety and depression, higher stress, and lower positive well-being. No effects of the intervention were observed for high-intensity features, regardless of participants’ mental health status at baseline.

Our findings show that an online, single-session growth-mindset intervention, when precisely designed to target a relevant psychological barrier, can meaningfully promote the successful initiation of MHA engagement among people who might otherwise be less likely to seek or persist with mental health support.

One possible explanation for this effect is that individuals with more clinically relevant symptoms may have a greater psychological need for change, which might increase their receptiveness to belief-shifting interventions. As shown in previous studies, individuals often perceive clinical symptoms to be more urgent, which can create a psychological state that heightens their motivation to seek relief^67^. When presented with the idea that mental health is malleable and can be improved through effort, they may be more likely to process the message deeply, recognize its personal relevance, and initiate seeking treatment. However, this proposed mechanism must be confirmed by future research.

Our study contributes to the growing body of research on wise interventions, which are brief, targeted interventions designed to modify specific psychological processes that may initiate recursive and lasting improvements in behavior^68^, as demonstrated by previous growth-mindset interventions^55,56,69–71^. Consistent with this framework, we targeted a specific belief that may undermine sustained engagement in mental health care, namely the belief that mental health is fixed and cannot be meaningfully improved. Based on previous research linking fixed mindsets to the avoidance of proactive behaviors for well-being^48–50^, we theorized that a fixed mindset of mental health would serve as an important psychological barrier to engaging with mental health resources, such as an MHA. Our intervention was designed to present the understanding that mental health is improvable and can be strengthened through intentional effort. After completing this treatment, participants in the growth-mindset condition showed a significantly higher likelihood of repeat engagement with self-help features on a digital mental health resource, relative to the control condition. We believe that these findings contribute to the literature on wise interventions by suggesting that a growth-mindset intervention can promote early treatment-seeking behaviors by initiating engagement on a MHA.

Our findings also speak to emerging directions in mindset science and wise interventions, particularly in response to recent calls to better understand “for whom” and under what conditions these interventions are most effective^69,72^. The long-term effects of mindset and other wise interventions hinge not only on shifting a targeted belief, but also on the affordances of the situation—that is, whether the surrounding environment supports or hinders the intervention’s intended effect—and on the needs of the person receiving the intervention. For instance, O’Keefe et al. (2023) developed a growth-mindset-of-interest intervention that encouraged incoming arts undergraduates to view academic interests as developable, not fixed. They found that the intervention was especially effective at boosting interest and achievement (i.e., grades) in math and science courses among students who did not initially have an interest in those subjects, but who nonetheless had opportunities to engage with those subjects^55^. Similarly, Paunesku et al. (2015) found that a growth-mindset-of-intelligence intervention had its strongest effects among students who were underperforming and at risk of dropping out^57^. These findings suggest that individual differences can play a powerful role in shaping the effectiveness of an intervention. In line with these studies and others, we found that employees who benefited most from our intervention were those experiencing greater mental health difficulties at baseline. The growth-mindset-of-mental-health intervention may have been particularly relevant to people with poorer baseline well-being. Importantly, they may have been supported by an environmental affordance: participants’ workplaces had made the Intellect app freely available to them, giving them an opportunity to act on the intervention’s message. Together, these personal and environmental conditions may have contributed to their increased likelihood of initiating self-help engagement on the MHA, an idea worth pursuing more deeply in future work.

The pattern of repeat engagement emerged only for the MHA’s low- and moderate-intensity features. Employees with higher perceived stress or lower positive well-being at baseline were significantly more likely to return to the app and engage with low-intensity features, such as mood tracking. Meanwhile, employees with poorer baseline mental well-being across all three measures—higher perceived stress, higher anxiety and depressive symptoms, and lower positive well-being—were significantly more likely to return to engage with moderate-intensity features, such as breathing techniques and thought-challenging exercises. Yet, we did not find a greater likelihood of repeat engagement with high-intensity features. One possible reason is that these features were more effortful and may not have matched participants’ immediate needs^73^. High-intensity features often demand a level of cognitive effort, time, and emotional investment that can feel overwhelming for individuals already experiencing significant psychological symptoms. For example, features like guided journaling or structured learning paths typically involve deep reflection of newly learned psychoeducational content, and skill application through writing, which may be particularly taxing for individuals struggling with their mental health. It is also possible that participants were less likely to engage with high-intensity features due to other concerns about confronting distressing emotions or perceiving a lack of short-term relief. In short, low- and moderate-intensity features may have felt more manageable and accessible than high-intensity features, and thus were more likely to be selected for repeat engagement.

In line with the Transtheoretical Model, which conceptualizes behavior change as a dynamic progression through five stages—precontemplation (not intending to change), contemplation (considering change), preparation (making small changes), action (actively engaging in new behavior), and maintenance (sustaining change over time)^74,75^—it is possible that the growth-mindset-of-mental-health intervention motivated participants to progress from precontemplation to preparation. This may help explain why the treatment showed higher initiation of engagement with lower- and moderate-intensity features, where the activities may have felt more feasible, but did not facilitate progression to more effortful high-intensity features that may correspond more closely to the action stage. Given that wise interventions, by targeting a specific fixed belief, can set in motion recursive processes that accumulate and amplify gains over time^76–80^, it is also possible that a three-month window was insufficient to observe further progression toward engagement with more intensive features. Alternatively, because participants were not drawn from a more severely distressed clinical population, there may have been less readiness or need to progress to more advanced stages of change. Future research could test these possibilities directly by assessing stages of change before and after intervention exposure, and by examining whether booster sessions would facilitate greater progression toward higher-intensity engagement over time. That said, in the present study, we did not evaluate whether engagement with these features led to subsequent improvements in mental health outcomes. Future research should also incorporate follow-up assessments to test whether repeat engagement with these evidence-based features leads to subsequent improvements in well-being.

From a practical standpoint, our findings carry important implications for mental health care delivery. While MHAs offer a promising avenue for expanding access to evidence-based mental health support, particularly for individuals who lack the time and resources to pursue in-person care, their potential continues to be significantly limited by persistently low engagement rates. Our findings suggest that existing efforts to promote engagement (such as technical enhancements^16,27,28^) may not be sufficient on their own to drive engagement. Instead, our research suggests that mindsets may play a critical role, at least in early-stage engagement. We argue that replacing the view that mental health is unchangeable with the belief that it is improvable may be an underutilized strategy for increasing repeat engagement with MHAs. It is possible that this mindset shift may serve as a motivational gateway that technical features alone cannot unlock. However, because we did not assess growth mindset change post-intervention, we cannot determine whether changes in mindset mediated the observed increase in repeat engagement with app features. Future research would benefit from longitudinal designs that monitor changes in mindsets about mental health over time and examine whether these changes predict subsequent patterns of app engagement. Nonetheless, our study is among the first to apply growth mindset theory to mobile app engagement, and also one of the very few to examine help-seeking using objective behavioral data.

The present study has several notable strengths. First, our primary outcome relied on objective behavioral engagement data rather than self-reported intentions or retrospective recall of engagement behaviors. This is important because prior research on a growth mindset related to well-being primarily examined self-reported openness to lifestyle change, rather than objective engagement with mental health support^51^. Numerous digital mental health interventions also rely on self-reported usage, which may be easily influenced by recall bias or social desirability^81^. By directly tracking participants’ app use, the present study provides a more ecologically valid assessment of the effects of a growth-mindset-of-mental-health intervention on initiating real-world help-seeking behavior.

Second, we examined moderation of intervention effects by baseline mental well-being, which enabled us to identify for whom the intervention was most effective. This focus on heterogeneity aligns with growing calls within the behavioral sciences to move beyond average treatment effects and to examine how interventions may be adapted to specific populations and contexts^69,72^.

Finally, the growth-mindset-of-mental-health intervention increased repeat self-help engagement within a predominantly Asian sample, a culture where mental health stigma remains relatively high^82^. Observing effects in this culturally constrained help-seeking environment suggests potential applicability beyond this specific setting, although the extent to which the intervention generalizes to cultures that are more open to mental health treatment remains an empirical question for future research.

Several limitations should also be noted. First, our app engagement data were collected over a 3-month period, which provides a meaningful snapshot of short-term behavioral use but does not capture longer-term engagement trajectories. Although it is notable that 38.4% of our participants in the growth-mindset condition showed repeat engagement, particularly in light of prior evidence that as few as 20% of digital mental health app users return after an initial login^19^, this finding does not indicate whether this early engagement would be sustained over time. As such, it remains uncertain whether the observed effects of the intervention would persist, intensify, or diminish with time. Given that wise mindset interventions have been theorized to initiate recursive processes that can amplify over time^68,77,83^, future studies should incorporate extended follow-up periods to assess long-term usage patterns.

Second, although our primary outcome was based on objective behavioral data, we were unable to analyze mental health outcomes longitudinally. It remains an open question whether increased and sustained engagement with the app translates into clinically meaningful improvements in mental health. Prior trials of the Intellect app have demonstrated such benefits^59,61^, so future research should examine whether behavioral engagement serves as a mediator between mindset interventions and improvements in psychological well-being.

Third, the study did not include a formal post-intervention manipulation check assessing growth mindset change. As a result, we cannot directly test whether the growth-mindset-of-mental-health intervention altered the targeted belief, nor whether any change in mindset mediated subsequent engagement behavior on the app. Although the intervention was designed to promote a growth mindset of mental health, alternative explanations cannot be ruled out, such as the possibility that the intervention increased participants’ attention to mental health content, which in turn may have led to re-engagement with Intellect’s self-help features. Future studies should include post-intervention assessments of growth mindset and conduct mediation analyses to clarify the psychological mechanisms through which belief-shifting wise interventions influence engagement with MHAs.

Fourth, our engagement outcome captured only whether participants returned to use the app beyond completing an initial self-help feature, and did not assess the frequency, duration, or depth of engagement with the app’s content. As a result, we cannot draw conclusions about the quality of engagement, such as the extent to which participants actively practiced or applied the skills provided by the app, or whether such engagement translated into improvements in mental health over time. Future studies can consider collecting more fine-grained app engagement metrics (i.e., time spent on each feature) alongside follow-up assessments of mental health outcomes to better understand how different forms of engagement relate to treatment benefits. Specifically, with these additional data, future researchers could test the full mechanism proposed in this study: whether exposure to the growth-mindset intervention leads to a stronger growth mindset of mental health; whether such changes increase the initiation of app engagement; whether early repeat engagement develops into sustained engagement; and ultimately, whether this process contributes to improvements in mental well-being over time.

Lastly, the generalizability of these findings should be considered in light of the study sample and context. The intervention itself (e.g., the reading and writing activities) may depend in part on participants’ educational attainment, and initiation of app engagement may also be influenced by digital literacy or familiarity with technology. The sample consisted primarily of employed adults with relatively mild symptoms on average and higher educational attainment. As such, it remains uncertain whether these findings would generalize to more severely distressed clinical populations, to individuals with lower educational attainment or digital literacy, or individuals without access to employer-supported digital mental health tools.

This study demonstrates that a brief, scalable growth-mindset-of-mental-health intervention can increase repeat engagement with a mental health app, particularly among those who may need support most. By targeting users’ beliefs about the changeability of mental health, this intervention offers a novel and promising strategy to address a persistent challenge in digital mental health: helping people successfully initiate treatment and support.

## Method

### Transparency and openness

We report how we determined our sample size, the statistical software used, the analyses conducted, the statistical findings, and all measures included in this study. We follow standards for reporting psychological research in scientific journals^84^. This information and the research materials are available at the study’s Open Science Framework repository (https://osf.io/nsw2t/overview).

This study received ethical approval from the Institutional Review Board of National University of Singapore (ID: NUS-IRB-2022-253). The trial was conducted in full compliance with ethical standards, including the Declaration of Helsinki. All participants provided informed consent prior to their involvement in the study. We registered the study protocol at ClinicalTrials.gov (Identifier: NCT07071311) on 07-25-2025, to provide a public record of the trial and promote transparency. Because registration occurred after the study began, this study was not preregistered.

### Study design

We conducted a randomized controlled trial (RCT) to test the effectiveness of a brief intervention promoting a growth mindset of mental health, as compared to an active control condition that promoted the benefits of digital technology in the workplace. An active control condition was chosen as a conservative comparison, consistent with prior growth mindset RCTs, in which active controls are widely regarded as the gold standard in social-psychological intervention research^39,55,56,85,86^. The active control condition ensures that participants in both conditions engage in similarly-structured activities that vary only in their content, which helps control for expectancy effects and attention-related confounds. We tested whether the intervention would lead participants with lower baseline well-being—characterized by higher stress, anxiety and depression, or lower positive mental well-being—to show repeat engagement with self-help features on the *Intellect* app.

### Procedure

**Fig. 4:**
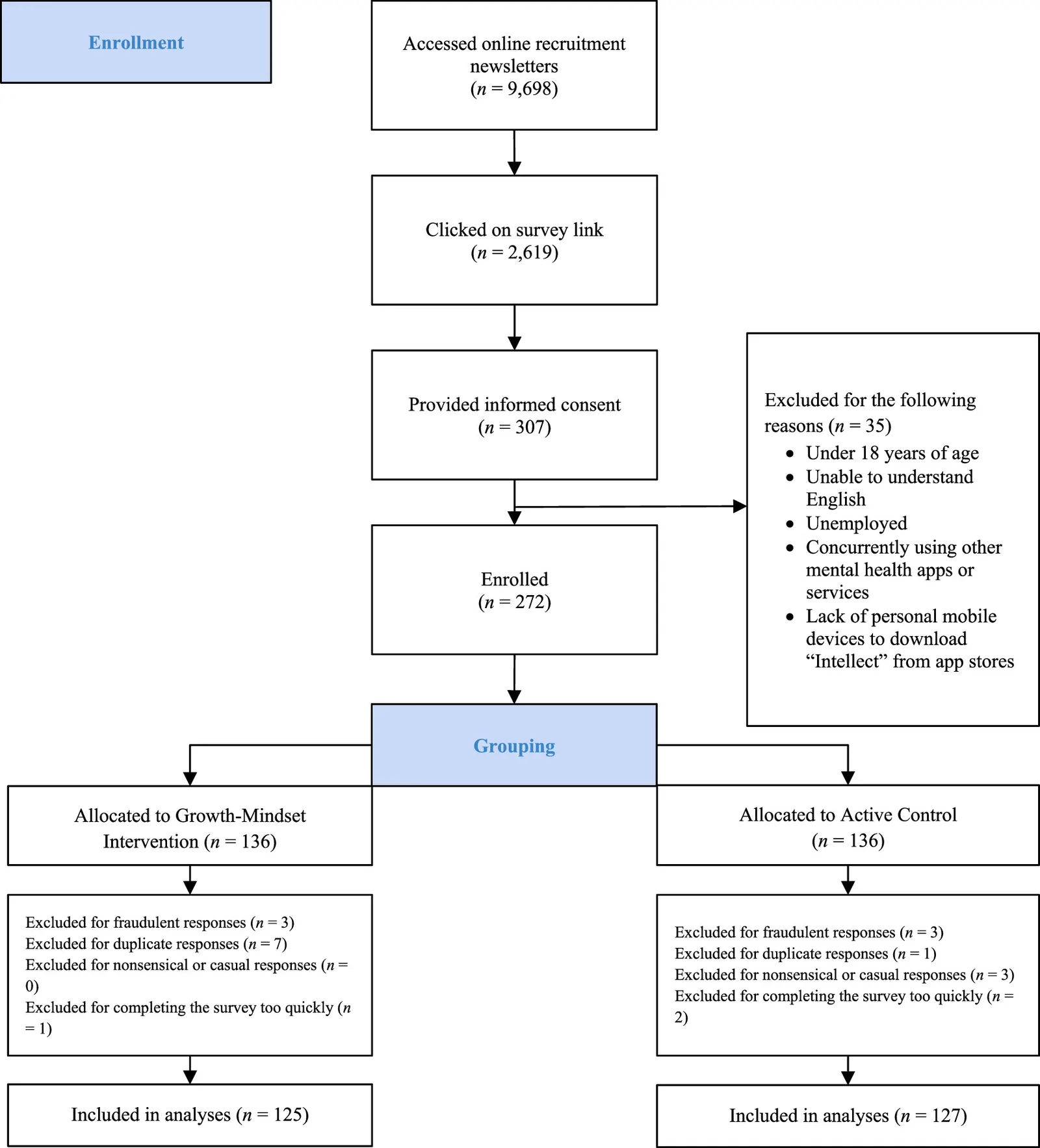
Participant CONSORT. The flow chart details participant recruitment, allocation, and retention across the study.

Recruitment took place from March to September 2023. Employees from 83 organizations received an email newsletter coordinated by Intellect’s client relations teams in collaboration with the human resource managers from their respective organizations. This email provided a brief overview of our research study, along with instructions on how to download and register for an account on the Intellect app. The final section of the newsletter included a link to a Qualtrics survey, where employees could access the Participant Information Sheet (PIS) and provide their informed consent to participate. According to Intellect’s internal data, 9698 employees opened the newsletter. Of those, 2619 clicked on the survey link and reviewed the PIS, which outlined the study’s aims, eligibility criteria, data protection measures, participant rights, investigator contact details, and study incentives. The aims of our study were partially concealed, as employees were told that the purpose of the study was solely to examine the use of a mobile health application (“Intellect”) in the workplace. This partial concealment was necessary to avoid demand effects, where expectations of a desired behavior can lead to improvements in that behavior^87^. A total of 307 employees provided informed online consent to participate. After providing consent, employees completed pre-screening measures. Inclusion required participants to report (a) being able to read and understand English, (b) being 18 years or older, (c) being employed, and (d) not using other mental health mobile applications or services concurrently. Thirty-five participants were excluded for failing to meet any one of these eligibility criteria (see Fig. 4).

The remaining 272 participants proceeded to complete a demographic questionnaire and three baseline mental well-being assessments: (a) anxiety and depressive symptoms, (b) perceived stress, and (c) positive mental well-being. Additional measures unrelated to the state of participants’ mental health (e.g., self-stigma of seeking professional help, social support, marital status) were also collected and are detailed in Supplementary Material. Participants were then randomly assigned by Qualtrics to one of two conditions (i.e., growth-mindset intervention or active control); thus, participants and researchers were blind to treatment condition. After completing the treatment materials, participants were given a SGD $10 online gift card. Participants then either created a new Intellect account (*N* = 149, 59.1%) or signed into a dormant account they created at an earlier time (*N* = 103, 40.9%). The latter indicates that 40.9% of participants had previously downloaded Intellect before the study. Compensation was not contingent on downloading the app or using any app features during the 3-month follow-up period. Participants were also not instructed to complete any specific app features after the baseline session.

All responses to the treatment materials were reviewed for quality in four ways, which resulted in several exclusions (see Fig. 4). First, we excluded responses identified as likely bots by Qualtrics’ reCaptcha scores (i.e., scores under .50; see www.qualtrics.com/support/survey-platform/survey-module/survey-checker/fraud-detection). Second, we removed duplicate responses based on repeated email addresses. Third, survey completion times were reviewed. Based on our pilot testing, which showed an average treatment completion time of 10 min, and recommendations to exclude responses completed in less than 40% of the average completion time^88^, any responses submitted in under 4 min were excluded. Finally, participants who left all three writing activities in the treatment materials entirely blank or provided nonsensical or non-response answers (e.g., blank, “NA,” “Yes,” “Don’t know”) were excluded, as this was evidence of inattention. The final sample consisted of 252 participants. No participants were excluded based on their subsequent app use or repeat-engagement outcomes.

### Treatment materials

This single-session growth-mindset intervention was developed using methods from the family of “Wise Interventions”^68,83,89^. It involved reading and reflective writing activities that portrayed mental health as improvable rather than fixed. To avoid eliciting demand, reactance, or stigmatization, the materials were not presented as an intervention or as addressing a deficit in employees. Instead, they were framed as part of an effort to gather insights to better support employees as a whole and improve their mental health. Participating employees were thus positioned as experts and co-creators of an intervention for other employees^77,90^.

Participants first read stories of three employees who shared their experiences overcoming mental health challenges in the workplace. Each described their struggles with workplace issues and their journey toward understanding that mental health is changeable, which includes taking proactive steps to improve it (e.g., deliberate self-care, or seeing a therapist). The stories represented diverse industries and professional roles, allowing participants to connect with a range of experiences. Since employees are likely to perceive their peers as credible and important sources of information, reading about how those peers proactively managed their mental health in response to workplace challenges may be particularly persuasive. This aspect of the material leveraged informational social influence^91^, as individuals often look to peers for guidance when forming beliefs and making decisions about their well-being. For example, one story illustrated how a director at a technology company suffering from anxiety attacks came to understand that his mental health was improvable and learned techniques to manage the anxiety after attending sessions with a clinician.

Next, participants wrote a brief essay reflecting on a time they successfully took action to improve their mental health while facing workplace challenges. They responded to the following prompt:

Take a moment and recall a time in your life when your mental health was poor but you took actions to improve your mental health. What did you do? In what ways did it improve your mental health and well-being?

They were informed that their essays would be used to support future employees in coping with mental health challenges at work. This activity leveraged the “saying-is-believing” effect, a technique whereby people advocate for a position to a relevant audience (i.e., other employees, in the present case), which strengthens their own endorsement of that belief^92^. By articulating that mental health is malleable and linking this idea to their own experiences, participants can come to endorse the belief more strongly for themselves (see Supplementary Material for more sample responses). As a representative example, one participant wrote:

People who believe that mental health can improve are more likely to take proactive steps toward improving it. Without the belief that change is possible, there’s little motivation to take action. This is why adopting the right mindset is essential.

Next, employees completed two additional brief essays, which again leveraged the saying-is-believing effect (see Supplementary Material for essay examples). In these essays, they offered advice to help other employees manage their mental health in response to the following prompts: “How might this understanding that mental health can improve help people with their own mental well-being, especially when work becomes stressful?” and “What can people do to improve their mental health when experiencing difficulties at work?” While writing, participants were also encouraged to draw on both the stories of other employees and their own experiences. By positioning participants as experts, helping to advise other employees at their company, the activity aimed to further strengthen their personal endorsement of a growth mindset^68^.

This control exercise was identical in approach and structure but instead promoted the use of technology in the workplace^93^. Unlike the intervention, the control exercise did not address mental health or its potential for development. Similar to the intervention, however, the control materials positioned employees as experts offering guidance to other employees, rather than as recipients of an intervention who were in need of help. These materials also emphasized a growth-oriented process, specifically focusing on the development of expertise in workplace technology as a crucial skill for overcoming challenges in the workplace. Therefore, these materials provided a rigorous comparison to test the effects of the intervention condition.

Participants first read stories about employees’ experiences managing their workload using workplace technology. Each described their challenges with learning and adapting to digital tools but ultimately came to see the value in technology use at work. This included viewing learning digital tools as manageable, and taking proactive steps, such as consulting with colleagues or making time to practice and improve. Different industries and roles were represented to resonate with participants’ own experiences at work.

Next, control participants wrote a brief essay about their personal experiences using digital tools to enhance their efficiency at work (see Supplementary Material for more sample responses). In response to the prompt “Take time to think about the digital technology you love most at work. What is the digital technology you use, and how does it help you?”, one representative response stated:

The growing adoption of online collaboration tools, such as Microsoft Teams and Microsoft Sharepoint, has greatly enhanced flexibility and efficiency. These tools allow us to communicate and collaborate on documents virtually at our convenience, enabling contributions to work without sacrificing personal time—This is an especially valuable benefit when working in a global organization. This aspect of digital technology is something I truly appreciate, as it has significantly supported me in my work.

After this reflection, participants in the control condition completed two additional essays, for which they were asked to offer advice to others on how to effectively use new technologies in the workplace. Their responses were guided by the following prompts: “Next, how do employees typically discover digital technologies at work?” and “How do employees apply those newly-discovered technologies at work?” Similar to the intervention materials, control participants were encouraged to draw from the employee stories they read and their own experiences with workplace technology.

### Measures

Following past mindset intervention research, we collected participants’ age and gender for use as covariates because these variables have previously been associated with mindset-related outcomes^56,94^. (Additional demographic and baseline variables not used in present analyses include employment status, highest level of education attained, marital status, occupational position, length of time in their current employment role, prior app usage, and baseline growth mindset of mental health. Controlling for these additional factors did not change the significance or pattern of any of our findings).

Participants’ baseline (pre-intervention) anxiety and depressive symptoms were assessed with the widely used 4-item Patient Health Questionnaire (PHQ-4)^95^, which has demonstrated excellent internal consistency and convergent validity^96^. Two items assessed depressive symptoms over the past 2 weeks (e.g., “Over the past 2 weeks, how often have you been feeling down or hopeless?”), and the other two assessed anxiety levels (e.g., “Over the past 2 weeks, how often have you felt nervous, anxious or on edge?”). Each item was rated 0 = *Not at all* to 3 = *Nearly every day*. Higher PHQ-4 scores indicate more severe anxiety and depressive symptoms. The cutoffs for mild, moderate, and severe anxiety and depressive symptoms are 3, 6, and 9, respectively. The Cronbach’s alpha for PHQ-4 in our current sample was high (α = 0.799).

Participants’ baseline stress levels over the last month were assessed using the 4-item Perceived Stress Scale (PSS-4)^97^, which has good convergent validity with other aspects of mental well-being^98^. A sample item is “In the last month, how often have you felt that difficulties were piling up so high that you could not overcome them?” (1 = *never* to 5 = *very often*). Higher scores reflect more perceived stress. The Cronbach’s alpha in our sample was good (α = 0.775).

Participants’ baseline positive well-being was assessed using the 7-item Short Warwick-Edinburgh Mental Well-being Scale (SWEMWBS)^99,100^, which has demonstrated good internal validity and test-retest reliability^101,102^. Sample items include “I’ve been feeling optimistic about the future” and “I’ve been feeling relaxed” (1 = *none of the time* to 5 = *all of the time*). Scores are summed, ranging from 7 to 35, with higher scores indicating more positive well-being. The Cronbach’s alpha in the current sample was high (α = 0.876).

### Engagement with app self-help features

Repeat app engagement was operationalized as the completion of at least one additional self-help feature beyond the first feature completed. This outcome captures whether participants returned to engage with another self-help feature on the app after completing an initial feature, but does not capture sustained or long-term engagement with the app. Nevertheless, completing another self-help feature reflects a more intentional and proactive effort to improve mental health. In contrast, using only a single feature is often interpreted as casual or exploratory use, rather than as evidence of successful initiation of self-help app engagement^103–106^. In this study, we assessed repeat engagement within 3 months of completing all assigned condition materials. Additionally, as secondary analyses, we assessed this with respect to distinct types of self-help features: low-, moderate-, and high-intensity. We also report app-use metrics for the overall sample in Table S2 of the Supplementary Material, including the types and average number of completed self-help features during the 3-month follow-up period.

Intellect’s full range of guided self-help features include “Mood and Stress Monitoring,” “Daily Toolkits,” “Rescue Sessions,” “Guided Journaling,” and “Learning Paths.” These features are categorized into three levels of intensity based on engagement time. Low-intensity features require relatively little effort and take less than 1 min, which include “Mood and Stress Monitoring.” Moderate-intensity features (“Daily Toolkits” and “Rescue Sessions”) require more effort and about 2 to 5 min to complete. Finally, high-intensity features (“Guided Journals” and “Learning Paths”) require the most effort and at least 10 min of focused engagement, including activities like writing and reflection. All three types of features on Intellect have been demonstrated to improve mental well-being in previous randomized controlled trials^58–64^.

“Mood and Stress Monitoring” allows users to log their current mood or stress levels. Users record their mood by sliding a scale and selecting from emojis labeled “terrible,” “not good,” “content,” “good,” and “happy.” A similar scale is used for stress, with options ranging from “very stressed” to “not stressed at all.” After logging their entries, users can view their previous moods and stress levels in a calendar format, allowing them to track emotional trends over time and gain insights into their emotional patterns. In line with recent studies^107,108^, mood-tracking features have been shown to help users identify patterns and potential causes of mood shifts, enabling them to take more informed actions to improve their well-being. We operationalized repeat engagement with low-intensity features by logging mood and stress levels at least twice (29.4% of the sample showed repeat engagement of low-intensity features).

“Rescue Sessions” are designed to alleviate in-the-moment anxiety through mindfulness and cognitive restructuring exercises. These sessions target common challenges, such as overthinking, high stress, low resilience, and burnout, and users may choose sessions that address their immediate concerns. Such challenges are often sustained by negative thought patterns^109^. Similar to CBT and mindfulness, “Rescue Sessions” guide users to either challenge the validity of their thoughts through Socratic questioning^110,111^ or to observe their thoughts from a distance and in a non-reactive manner^112,113^. Each session concludes with a recommendation for users to apply these techniques in their daily life.

Similarly, “Daily Toolkits” are brief mindfulness-centered features aimed at promoting a structured self-care routine throughout the day while addressing any negative thoughts that may arise. The morning segment consists of deep breathing exercises to focus on the present moment. Grounding techniques are introduced in the afternoon segment to help maintain mindfulness. Finally, the night segment promotes restful sleep through meditation and cognitive restructuring techniques^114^. We operationalized repeat engagement with moderate-intensity features by completing at least two “Rescue Sessions,” or “Daily Toolkits,” or at least one of each (23.4% of the sample showed repeat engagement of moderate-intensity features).

“Learning Paths” are self-guided, structured activities that provide in-depth psychoeducation and CBT-based self-help for various mental health problems. These features begin with an introduction to CBT principles, followed by guidance to enhance self-awareness of thoughts. Users then write about their own maladaptive thought–action cycles before receiving additional guidance to cognitively reframe these dysfunctional patterns. As an example, the “Feeling Depressed” learning path teaches users about common cognitive distortions, such as all-or-nothing thinking or overgeneralization^115^. Through structured exercises, users then reflect on their own distorted beliefs and write more balanced, evidence-based statements to reframe these negative thoughts.

“Guided Journals” prompt users to reflect on specific themes by recording their thoughts and then guiding them through cognitive restructuring techniques to reframe negative reflections^116^. These journals cover a broad range of themes, from challenges like ‘anxiety and depression’ and ‘poor self-esteem,’ to proactive improvements like ‘goal-setting’ and ‘sleep improvement,’ to maintaining mental well-being by highlighting positive factors (e.g., gratitude for positive events that occurred during the day). We operationalized repeat engagement with high-intensity features by completing at least two “Learning Paths” or “Guided Journals” or at least one of each (29.0% of the sample showed repeat engagement of high-intensity features).

### Analytic approach

All analyses were conducted in SPSS V29.0. To test hypotheses, we used the PROCESS macro V.4.2. (Model 1) in SPSS^117^ to examine whether baseline well-being variables (anxiety and depression, perceived stress, or positive well-being) moderated the effect of treatment condition on the likelihood of repeat engagement of self-help features. Continuous independent and moderating variables were mean-centered. Following past mindset intervention research^55^, age (in years) and gender (1 = *female*, 0 = *male*) were included as covariates. Overall repeat engagement with self-help features was coded as a binary variable (0 = *no repeat engagement*, 1 = *repeat engagement*). Additionally, repeat engagement with each type of self-help feature (low-, moderate-, or high-intensity) was coded as binary (0 = *no repeat engagement*, 1 = *repeat engagement*). Since the outcome variables are binary, logistic regression was used for analyses.

For our primary analysis, we tested the interaction of condition and baseline well-being on overall repeat engagement. Because we had three distinct baseline well-being moderators, we conducted a separate model for each one (totaling three central models). Subsequently, to better understand which type of features drove our primary effects, we conducted those same three models separately for low-, moderate-, and high-intensity self-help features. Statistical significance was set at α = 0.05. Controlling for the age and gender covariates did not change the significance or patterns of our findings.

## Supplementary information

Supplementary information

Supplementary information

## Data Availability

The data analyzed in this study are not publicly available due to restricted permissions and confidentiality shared between Intellect and its users. The data can be made available upon reasonable request to the corresponding author.

## Code availability

No code was developed for this study. All statistical analyses were conducted using IBM SPSS Statistics (Version 29). All outputs are available at the study’s Open Science Framework repository (https://osf.io/nsw2t).
