# Supplementary material for: A “growth mindset of mental health” intervention increases the likelihood of initiating self-help on a mental health app: a randomized controlled trial
Source: NPJ Digit Med. 2026 Oct 13;9:806. doi: 10.1038/s41746-026-03322-w (PMC13691065; doi:10.1038/s41746-026-03322-w)
Supplement: Supplementary file 1 [file 41746_2026_3322_MOESM1_ESM.pdf]

Supplementary Material

Sample Responses from the Growth Mindset Writing Activities

Prompt: Take a moment and recall a time in your life when your mental health was poor but you took actions to improve your mental health. What did you do? In what ways did it improve your mental health and well-being?

"People who believe that mental health can improve are more likely to take proactive steps toward improving it. Without the belief that change is possible, there's little motivation to take action. This is why adopting the right mindset is essential."

- Employee A

"When I first started out working in the mental health sector, I absolutely loved the many diverse interactions I had in a day. After a few months, though, I realized that centering my thoughts around other people's problems and other people's topics for 8+ hours each day drained me. I often came home, not ready to interact with my family because I felt like I couldn't take on any additional information, complaints, or stress. I noticed that I started withdrawing from my family in order to gain time for myself, which wasn't what I wanted for myself. By starting therapy, I finally had a weekly, scheduled time that was just about ME and my problems, and my accomplishments. It gave me the space to focus on myself and thereby the strength to focus on everybody else during the day."

- Employee B

"I became depressed after a significant decision was made against my wishes which irrevocably changed the course of my life. I lost all my friends from the long bout of reclusiveness. It helped

that I started a new hobby, and found fame and friends online by sharing my works. I also had the good fortune of coming across a psychodynamic therapist who, although still a student, was very good at therapy. It did not cure my depression, but it opened up a new dimension of introspection for me, and this improved ability to introspect helped me cope with my feelings."

- Employee C

Prompt: How might this understanding that mental health can improve help people with their own mental wellbeing, especially when work becomes stressful?

"It brings one's locus of control back towards the internal. This helps individuals realise that although there are work externalities that they cannot control, they can change how they react to it. With more adaptive responses and coping mechanisms, people can improve their own mental wellbeing when faced with work stress."

- Employee D

"When people understand that mental health can improve, they may be more likely to seek help when they experience stress or other mental health challenges. They may feel more confident in seeking support from a therapist or other mental health professional, and more open to trying different types of treatments or interventions."

- Employee E

"Understanding that mental health can improve is an essential factor that can help people with their mental wellbeing, especially during times of stress, such as work-related stress. Knowing that mental health is not a static state and can be improved can provide hope and motivation to

individuals struggling with mental health challenges."

- Employee F

Prompt: What can people do to improve their mental health when experiencing difficulties at work?

"For starters, people should learn about mindfulness. They need to learn more about their own emotions and to recognize them as and when they are happening. Then, they need to learn techniques on how to deal with certain emotions like meditation, breathing exercises, etc. If they have the opportunity, then they can also go talk with a professional (eg. a therapist, psychologist, a coach, etc. depending on the situation). They can also discuss their situation with others who have experienced similar difficulties in a safe environment, but I suspect this one is very difficult to achieve."

- Employee G

"Do something which they enjoy instead of worrying over work"

- Employee H

"To improve mental health when experiencing difficulties at work, individuals can practice self-care, set boundaries, seek support, use stress management techniques, prioritize self-reflection, take breaks, communicate openly, and engage in activities outside of work for relaxation and rejuvenation."

- Employee I

Sample Responses from the Active Control Writing Activities

Prompt: Take time to think about the digital technology you love most at work. What is the digital technology you use, and how does it help you?

"SQL is a powerful language that lets me extract data in the format I need. This technology helps me save lots of time on data scrubbing and focus more on analytics. The time saved helps me become more productive overall."

- Employee J

"I've been using ChatGPT extensively in the past few months. It's a great and powerful product, which made my life much easier at work."

- Employee K

"I personally use Asana to keep on top of what I have to do every single day. As someone who is basically a scatterbrain and shifts between tasks constantly, Asana is amazing for allowing me to put down notes on what I'm left to complete, and when I have to complete them by. It's easy to use, and I can easily see everything I have to achieve/complete within a certain period. It also sends me reminders when I choose to have it on!"

- Employee L

Prompt: Next, how do employees typically discover digital technologies at work?

"One way is through word of mouth, where colleagues or supervisors may recommend GitHub as a tool for code management and collaboration. Another way is through onboarding or

training sessions, where new employees may be introduced to GitHub as part of the organization's development process. Additionally, developers may have already been familiar with GitHub from previous jobs or their personal use and bring it up as a suggestion to their team or supervisor. In some cases, organizations may have a policy of using GitHub or similar tools for code management, in which case employees may be required to use it as part of their job responsibilities."

- *Employee M*

"Employees can discover digital technologies at work through various channels, including training and onboarding programs, internal communication channels, IT support and helpdesk services, colleagues and peers, self-exploration and learning, vendor or supplier training, and company policies and guidelines. It's important for companies to provide resources and support to help employees effectively learn and adopt new technologies in the workplace."

- *Employee N*

"Our IT team has been helpful at sharing tips. Among colleagues, we also recommend different tools to each other"

- *Employee O*

Prompt: How do employees apply those newly-discovered technologies at work?

"Employees typically experiment with new digital technologies by integrating them into their daily workflows and testing them out to see how they can enhance their work processes"

- *Employee P*

"Company-wide usage of technologies encourages faster adoption. Employees can learn from colleagues on how certain tools can be used to enhance productivity. Frequent-use can help build proficiency and habit-building."

- *Employee Q*

"I think it depends on how receptive employees are to new technologies - Some may find it hard to break their old habits and move to a new platform, even if it greatly helps to increase their productivity. Others may be more keen to adopt new platforms if they know of their pros."

- *Employee R*

Other Measures

Implicit Theories of Mental Well-Being (Baseline)

Prior to the randomization, participants' mindset about mental well-being was assessed using an 8-item Implicit Theories of Mental Well-being Scale adapted from Dweck (2013), which assessed mindsets about intelligence. Items were rephrased so that each statement reflected the degree to which mental health was fixed or malleable. Examples include "To be honest, you can't really change your level of mental well-being" and "Your mental well-being is something about you that you can't change very much." Each item was rated on a 6-point Likert scale, ranging from 1 (*strongly disagree*) to 6 (*strongly agree*). Items are reverse-scored such that higher scores reflect a stranger growth mindset.

Absenteeism

Absenteeism was assessed using an item from the Work Productivity and Activity Impairment Questionnaire (WPAI; Reilly et al., 2004). The item asks participants to indicate the number of days they missed work due to mental and physical health reasons (e.g., stress, anxiety, fatigue) over the last month. Higher scores indicate higher levels of absenteeism.

Presenteeism

Presenteeism scores were assessed using the Single-item Presenteeism Questionnaire (SPQ; Muramatsu et al., 2021). Participants rated their perceived job performance over the past month on a 100-point scale. Higher scores indicate greater engagement at work (i.e., lower presenteeism).

Perceived Social Support

Participants' perceived social support levels were assessed using the validated, 6-item Brief Perceived Social Support Scale (Fragebogen zur Sozialen Unterstützung Kurzform mit sechs Items, F-SozU K-6; Kliem et al., 2015). Sample items include "If I need to, I can borrow

something from friends or neighbors without any problems" and "If I'm very depressed, I know who I can turn to." Each item was rated from 1 (*Not true at all*) to 5 (*Very True*). Higher scores reflect higher perceived social support. The F-SozU K-6 demonstrated strong reliability and convergent validity with both positive and negative well-being dimensions (Klim et al., 2015). The scale also revealed strong cross-cultural measurement invariance in multiple general population and student samples across countries such as the U.S., Germany, China, and Russia (Lin et al., 2019).

Self-Stigma Of Seeking Professional Help

Self-stigma of seeking professional help was assessed using the 10-item Self-Stigma of Seeking Help instrument (SSOSH) (Vogel et al., 2006). This instrument measures the extent to which people perceive a threat to their self-esteem if they were to seek professional counseling or psychotherapy services. Sample items include "I would feel inadequate if I went to a therapist for psychological help" and "I would feel inadequate if I went to a therapist for psychological help." Each item was rated from 1 (*Strongly Disagree*) to 5 (*Strongly Agree*), and higher scores reflect a stronger self-stigma towards seeking professional help for mental health. The SSOSH scale demonstrates strong internal consistency, with reliability coefficients ranging from .81 to .91 in community samples (Vogel et al., 2013). The scale also uniquely predicted attitudes toward seeking help (r = -.65) and intent to seek help (r = -.65) (Vogel et al., 2013).

Descriptive Analyses

Table S1

Descriptive Statistics for Baseline Variables by Condition

Variables		Growth-Mindset Condition (n = 125) M (SD) / n (%)	Active Control Condition (n = 127) M (SD) / n (%)	p-value
Age		33.01 (8.13)	31.95 (7.24)	0.277
Gender	Female	49 (39.2%)	63 (49.6%)	0.229
	Male	74 (59.2%)	63 (49.6%)	
	Others	2 (1.6%)	1 (0.8%)	
Highest Education Attained	Secondary School (Grade 10)	0 (0%)	4 (3.1%)	0.486
	Pre-University (Grade 12)	18 (14.4%)	16 (12.6%)	
	Bachelor's Degree	78 (62.4%)	78 (61.4%)	
	Master's Degree	26 (20.8%)	25 (19.7%)	
	Doctoral Degree	1 (0.8%)	2 (1.6%)	
	Professional Degree (MD)	2 (1.6%)	2 (1.6%)	
Marital Status	Married	41 (32.8%)	39 (30.7%)	0.378
	Married but Separated	2 (1.6%)	0 (0%)	
	Divorced	4 (3.2%)	2 (1.6%)	
	Never Married / Not Yet Married	78 (62.4%)	86 (67.7%)	
Occupation	Management Level	66 (52.8%)	48 (37.8%)	0.023
	Not In Management	59 (47.2%)	79 (62.2%)	
Positive Mental Wellbeing		22.9 (4.85)	23.2 (4.75)	0.592
Anxiety and Depressive Symptoms		7.92 (2.43)	8.00 (2.65)	0.803
Perceived Stress		11.0 (2.71)	11.1 (2.89)	0.825
Used Intellect Before	No	67 (53.6%)	82 (64.6%)	0.077
	Yes	58 (46.4%)	45 (35.4%)	

Table S2

Descriptive App-use Metrics for the Overall Sample (N = 252)

Category/Feature Type	*M* (*SD*)
Total Completed Self-Help Features	11.11
Low-Intensity Features	4.32
Mood Check-ins	0.96
Stress Monitoring	3.37
Moderate-Intensity Features	2.02
Rescue Sessions	0.71
Daily Toolkits	1.31
High-Intensity Features	4.78
Learning Paths	3.92
Guided Journals	0.84

Note. Completed self-help features are reported as mean frequencies. Metrics are reported for the final sample during the 3-month follow-up period.

Intellect's Guided Self-Help Features

Figure S1
"Self-Esteem" Learning Path

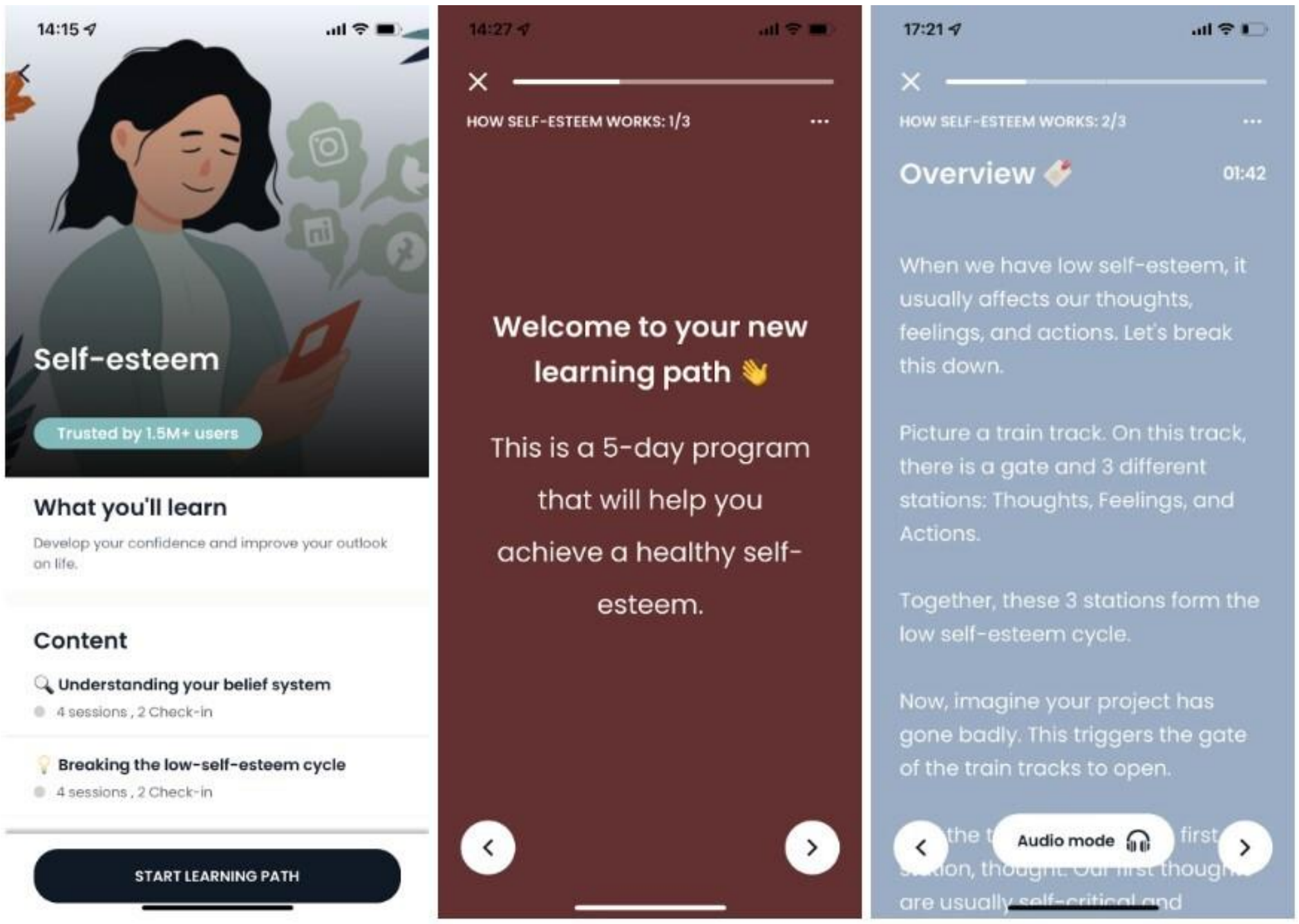

Figure S2
"Anxiety & Worry" Learning Path

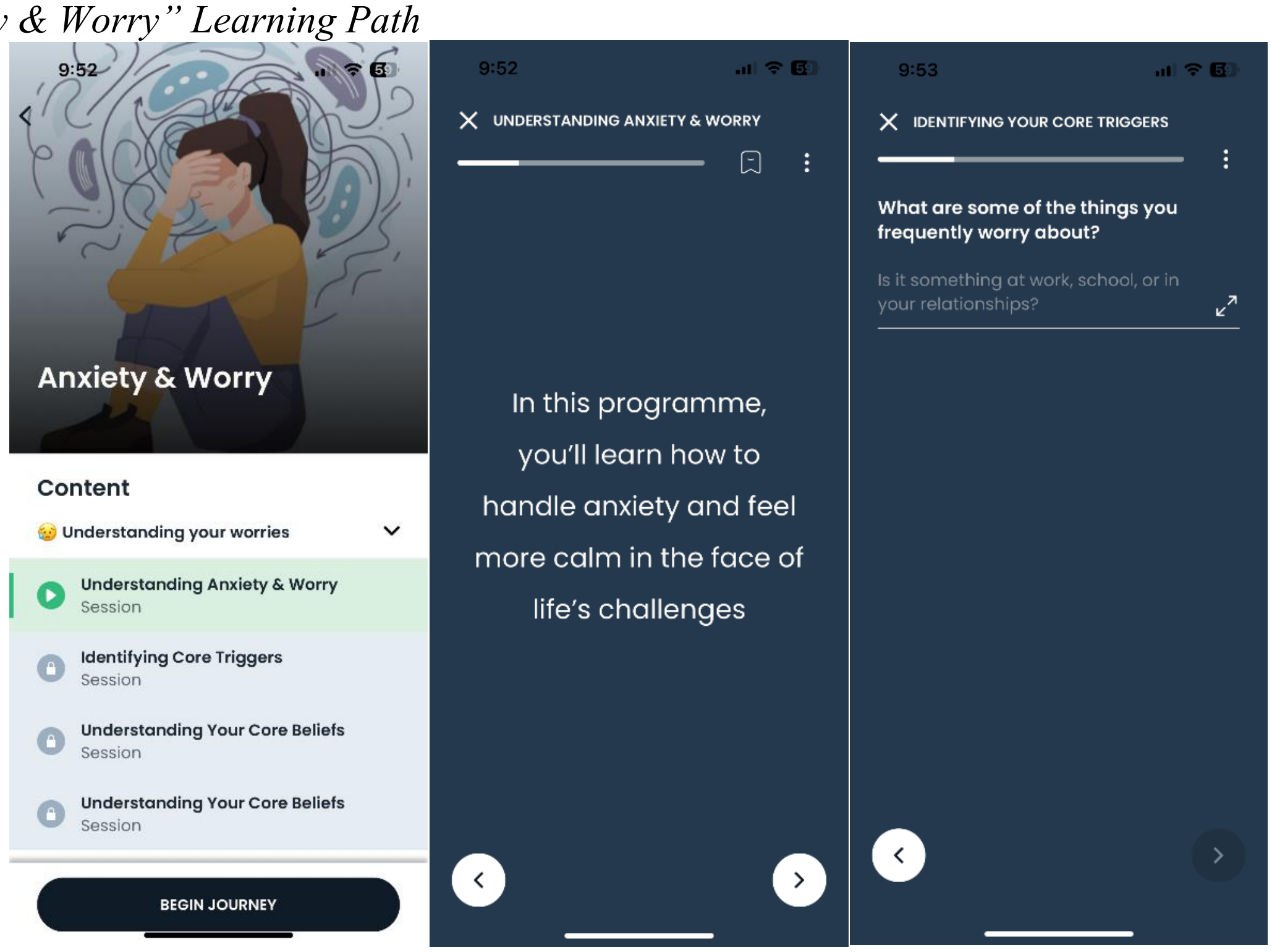

References

1. Dweck, C. S. (2013). Self-theories: Their role in motivation, personality, and development. Psychology Press.
2. Kliem, S., Mößle, T., Rehbein, F., Hellmann, D. F., Zenger, M., & Brähler, E. (2015). A brief form of the Perceived Social Support Questionnaire (F-SozU) was developed, validated, and standardized. *Journal of Clinical Epidemiology*, 68(5), 551–562.
3. Lin, M., Hirschfeld, G., & Margraf, J. (2019). Brief form of the Perceived Social Support Questionnaire (F-SozU K-6): Validation, norms, and cross-cultural measurement invariance in the USA, Germany, Russia, and China. *Psychological Assessment*, 31(5), 609.
4. Muramatsu, K., Nakao, K., Ide, H., & Furui, Y. (2021). Testing the construct validity and responsiveness of the single-item presenteeism question. *Journal of Occupational and Environmental Medicine*, 63(4), e187–e196.
5. Reilly, M. C., Bracco, A., Ricci, J. F., Santoro, J., & Stevens, T. (2004). The validity and accuracy of the Work Productivity and Activity Impairment questionnaire–irritable bowel syndrome version (WPAI: IBS). *Alimentary pharmacology & therapeutics*, *20*(4), 459-467.
6. Vogel, D. L., Wade, N. G., & Haake, S. (2006). Measuring the self-stigma associated with seeking psychological help. *Journal of Counseling Psychology*, 53(3), 325.
7. Vogel, D. L., Armstrong, P. I., Tsai, P. C., Wade, N. G., Hammer, J. H., Efstathiou, G., ... & Topkaya, N. (2013). Cross-cultural validity of the Self-Stigma of Seeking Help (SSOSH) scale: examination across six nations. *Journal of Counseling Psychology*, 60(2), 303.
